# Stable Operation of Paired CO_2_ Reduction/Glycerol Oxidation at High Current Density

**DOI:** 10.1021/acscatal.3c05952

**Published:** 2024-04-13

**Authors:** Attila Kormányos, Adrienn Szirmai, Balázs Endrődi, Csaba Janáky

**Affiliations:** Department of Physical Chemistry and Materials Science, University of Szeged, Aradi sq. 1, Szeged 6720, Hungary

**Keywords:** electrocatalysis, electrolysis, electrosynthesis, carbon capture and utilization, dynamic electrochemical protocols

## Abstract

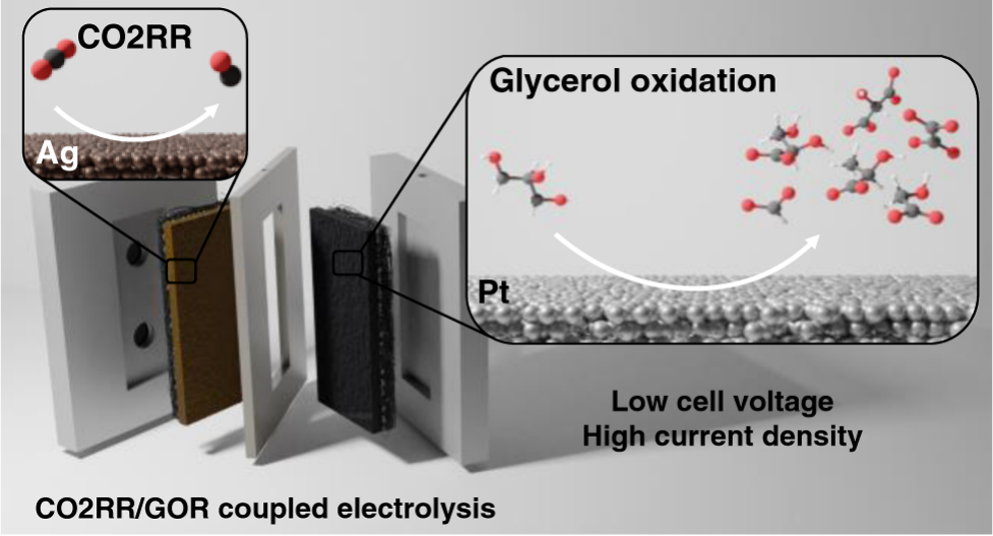

Despite the considerable efforts made by the community, the high operation cell voltage of CO_2_ electrolyzers is still to be decreased to move toward commercialization. This is mostly due to the high energy need of the oxygen evolution reaction (OER), which is the most often used anodic pair for CO_2_ reduction. In this study, OER was replaced by the electrocatalytic oxidation of glycerol using carbon-supported Pt nanoparticles as an anode catalyst. In parallel, the reduction of CO_2_ to CO was performed at the Ag cathode catalyst using a membraneless microfluidic flow electrolyzer cell. Several parameters were optimized, starting from the catalyst layer composition (ionomer quality and quantity), through operating conditions (glycerol concentration, applied electrolyte flow rate, etc.), to the applied electrochemical protocol. By identifying the optimal conditions, a 75–85% Faradaic efficiency (FE) toward glycerol oxidation products (oxalate, glycerate, tartronate, lactate, glycolate, and formate) was achieved at 200 mA cm^**–**2^ total current density while the cathodic CO formation proceeded with close to 100% FE. With static protocols (potentio- or galvanostatic), a rapid loss of glycerol oxidation activity was observed during the long-term measurements. The anode catalyst was reactivated by applying a dynamic potential step protocol. This allowed the periodic reduction, hence, refreshing of Pt, ensuring stable, continuous operation for 5 h.

## 1 Introduction

The electrochemical conversion of CO_2_ (CO2RR) is an attractive avenue to simultaneously decrease its emission and generate valuable platform chemicals.^1^ Thanks to intense scientific and industrial interest, remarkable progress has been made in this field during the past decade. This includes the demonstration of industrially relevant partial current densities for CO, C_2_H_4_, and HCOO^–^ production at the laboratory scale,^2−4^ along with the long-term (from hundreds to a few thousand hours) stable operation of continuous-flow electrolyzer cells,^3^ and the development of the first multilayer electrolyzer stacks.^5^ The scale-up of the CO_2_ electrolysis technology is currently in progress at several sites worldwide, targeting industrialization.^1,6^ Traditionally, the anode process coupled with CO_2_ reduction is the oxygen evolution reaction (OER), and most probably, this will be the case in the first generation of industrialized CO_2_ electrolysis technologies. At the same time, there is a significant gap between the standard redox potentials of CO2RR and OER, which, together with the sluggish kinetics of both reactions, results in high cell voltages (typically around 3 V).^7^ Therefore, it is important to seek alternative anode reactions that could substantially decrease the energy required for operating the electrolyzer cell. In addition, another factor to consider is the market value of the products formed at the anode.^8^ There are several requirements that an ideal reaction should fulfill, such as:

- The reaction can be driven at less positive potentials compared to the OER, even at high current densities.
- The market value of the formed product(s) is higher compared with the substrate molecules.
- The carbon-neutral/negative operation of the complete electrolyzer cell can be ensured.

There are several reactions that have already been considered, such as the electrocatalytic oxidation of chloride ions,^9^ aliphatic and aromatic alcohols,^10,11^ urea,^12^ amines,^9^ hydrazine, and other, biomass-derived compounds^13−17^ (most importantly, 5-(hydroxymethyl)furfural and glucose). A promising candidate is glycerol, a common byproduct of the soap/biodiesel industry (80% purity glycerol costs about 0.24 USD kg^–1^).^7^ It can be accessed in large quantities from a relatively pure source, and its electrocatalytic conversion to valuable products fits all the above-outlined criteria.^7^

The replacement of OER with electrocatalytic alcohol oxidation (AOR) was first attempted in conjunction with electrochemical hydrogen production, and there are numerous studies demonstrating the viability of this concept even in continuous-flow electrolyzer cells.^18−20^ Contrastingly, in the case of CO_2_ electrolysis, it has only gained momentum recently. Only a few studies can be found so far, and the vast majority of these were conducted in H-cell setups, and not in flow cells. Because of this infancy, the achievable current densities and long-term stability are-yet-far off from what could be pushed toward an industrial application trajectory.

The pool of applicable AOR catalysts can be divided into two groups, with both having their own advantages and disadvantages. The first is the nonnoble metal catalysts based on, for example, Co,^21^ Ni,^21−23^ etc. The common feature of these materials is that the onset potential of the AOR is above the oxidation potential of the catalyst surface (≈+1.10 V_RHE_); hence, AOR is most likely driven by radicals, just as in the case of the OER. Therefore, the decrease in the cell voltage (i.e., lower energy needs) of the paired CO2RR/AOR electrolyzer cell (compared to the OER scenario) is marginal at best. On the other hand, nonnoble transition metal electrocatalysts can be very selective in the glycerol oxidation reaction, forming mostly formate,^22^ which could eliminate/decrease additional downstream separation costs. The other option is noble metals as electrocatalysts, such as Pt,^7,24−26^ Au, Pd,^27,28^ and their multimetallic derivatives, PtRu,^29,30^ and PdAu.^27^ GOR can be driven at several hundred mVs less positive potentials compared to the OER.^7,31^ A major general issue with using noble metals in GOR is that, mostly, a complex mixture of C1–C3 products forms, mandating the addition of separation/purification step(s). For example. Pt in an alkaline environment typically generates a mixture of dihydroxyacetone, glyceraldehyde, glycerate, lactate, tartronate, glycolate, oxalate, and formate.^24,26,32,33^ Another major hurdle is that glycerol and the formed intermediates/products can irreversibly adsorb at the catalyst surface, (slowly) poisoning it.^33−35^ Finally, at reasonable current densities, GOR potentials fall in the potential window in which the catalyst surface starts to oxidize (*E*_ox,Pt_ ≈ +0.95 V_RHE_ according to the Pourbaix diagram)^36^ leading to the formation of a compact oxide layer,^37^ inactivating the electrode for GOR.^25^ Au is an exception in this sense since its oxidation starts above +1.20 V_RHE_ in alkaline electrolytes.^38,39^

As described above, the poor stability of noble metal catalysts in GOR stems from both the adsorption of strongly bound intermediates and from the partial/total oxidation of the electrocatalyst surface. Both issues can be successfully tackled by switching from the “static” (galvano-, or potentio-) electrochemical protocols to dynamic ones.^16^ Such strategies have been successfully applied for different anode,^16,40^ and cathode^41−44^ reactions. The goal of these methods is to periodically restore the initial surface conditions (e.g., remove accumulating reactants and products, restoring the oxidation state of the catalyst). The first examples of pulsed protocols steering the selectivity of CO2RR date back several decades.^45,46^ As a specific example, the potential was switched between −0.80 V_RHE_ and −1.15 V_RHE_ during CO_2_ electrolysis, applying sputtered Cu as the cathode catalyst.^43^ CO_2_ enriched at the Cu/electrolyte interface during the less negative pulse, modifying the ratio of the adsorbed CO/H at the catalyst surface, hence increasing the selectivity toward C_2+_ product formation.^42,43^ Besides CO2RR, an interesting application of such dynamic electrochemical protocols is organic electrosynthesis, where, for example, the enantiomeric yield increases in the stereospecific electroreduction of prochiral model molecules (acetophenone to 1-phenylethanol) at chiral mesoporous metal structures (Pt in the cited example).^47^ The turnover frequency was increased by orders of magnitude during the electrocatalytic oxidation of sorbitol, along with the notable increase of the ketoses-to-aldoses ratio (the secondary alcohol oxidation pathway becomes more favored) when the traditionally applied potentiostatic protocol was replaced by a potentiodynamic one applying Pt/C as the anode catalyst.^16^ An intermittent potential control strategy allowed the electrocatalytic oxidation of benzyl alcohol on a Au/CoOOH catalyst at high current densities (*j* ≈ 300 mA cm^–2^) for 24 h with constant selectivity (benzaldehyde and benzoic acid products).^41^ The formation of an oxide layer (either PtO_*x*_ or AuO_*x*_) was identified as the main phenomenon responsible for rapid performance fading during static electrochemical measurements. The dynamic pulsing of the potential (cyclic voltammograms in the case of Pt and shifting of the potential to the OCP for the Au/CoOOH sample) allowed the periodic reduction of the previously formed surface oxide layer, thereby preserving the activity of the electrocatalyst.

In this study, the conversion of CO2RR to CO was coupled with GOR in a membraneless microfluidic electrolyzer cell, employing carbon-supported Pt (Pt/C) as the anode catalyst. This system is a step forward from classical electrochemical setups (stagnant electrolyte, three-electrode H-cells) allowing us to study the effect of cell components (current collectors, gas diffusion layers, catalyst layers, etc.) and process conditions (CO_2_ and electrolyte flow rate, electrolyte composition and pH, glycerol concentration, etc.) on the achievable reaction rates, selectivity (of both the cathode and anode reaction) and the stability of the paired electrolysis process under continuous-flow conditions with relative ease (compared to a zero-gap setup). First, the anode catalyst layer composition and cell operating conditions were optimized. The initially observed gradual deactivation of Pt was circumvented by performing a dynamic electrochemical protocol, where a short potential pulse at reducing potentials was introduced periodically, preserving the activity and selectivity of the catalyst. Our results provide an important starting point in the endeavor of scaling up paired CO2RR/GOR processes.

## 2 Experimental Section

### 2.1 Chemicals

Potassium hydroxide (KOH, VWR), isopropanol (IPA, VWR), and glycerol (VWR) were of analytical grade and used without further purification. Carbon-supported Pt (Pt/C, on KetjenBlack, 60 wt % metal loading), iridium black (high surface area), and aqueous Nafion dispersion (10 wt %) were acquired from Fuel Cell Store, while Ag nanoparticles (*d* <100 nm) were purchased from Sigma-Aldrich. Aqueous Capstone ST-110 latex pore sealer fluoropolymer (CST) dispersion was purchased from Chemours. A 4.5 purity CO_2_ (Messer) and 4.7 purity Ar (Messer) were used to conduct the paired CO2RR/GOR electrolysis experiments. All electrolyte solutions were prepared using Type I grade water (Millipore Direct Q3-UV, 18.2 MΩ cm).

### 2.2 Assembly of Anode and Cathode Gas Diffusion Electrodes

All gas diffusion electrodes (GDEs) were prepared by spray-coating the (supported) catalyst nanoparticles on Freudenberg H23C6 (FRG H23C6) gas diffusion layers (GDLs), using an Alder AD-320 airbrush and compressed air (*p* = 0.6 bar). GDLs were placed on a hot plate preheated at *T* = 100 °C. Ag nanoparticles (Ag NPs) were used as the cathode catalyst. Ag NPs (196 mg) were dispersed in a mixture of 4 cm^3^ ultrapure H_2_O and 4 cm^3^ IPA. Prior to adding the CST, the dispersion was pretreated with a high-power sonotrode (Hielscher UP200ST). CST was added to the Ag dispersion (5 wt %) followed by a final homogenization step in an ultrasonic bath for 20 min. The bath temperature was always maintained below 35 °C. The spray-coated catalyst loading was 1 mg cm^–2^ (metal content).

Carbon-supported Pt NPs and iridium black were employed as anode catalysts. Both catalysts (150 mg) were dispersed in 3.75 cm^3^ ultrapure H_2_O and 3.75 cm^3^ IPA. Fifteen wt % Nafion was added to the Ir-containing dispersion, while the quality (either Nafion or CST) and quantity (0–15 wt %) of the ionomer were varied in the case of the Pt/C catalyst layers. Catalyst dispersions were homogenized for 20 min in an ultrasonic bath. The spray-coated catalyst loading was 1 mg cm^–2^ (metal content, not counting the mass of the carbon support/ionomer).

### 2.3 Morphological and Physical Characterization

Morphology and composition of the as-prepared and surface-modified anode GDEs were mapped with scanning electron microscopy (SEM, Thermo Scientific Apreo 2) equipped with an energy-dispersive X-ray detector.

The morphology of the Pt/C anode catalyst was further analyzed with transmission electron microscopy (TEM) before and after performing the “static” and “dynamic” long-term electrochemical protocols. Images were captured using an FEI TECNAI G2 20 X-Twin TEM with an accelerating voltage of 200 kV. All samples were prepared on a lacey carbon support.

The crystal structure of the catalysts was studied with X-ray diffraction (XRD) using a Rigaku MiniFlex II instrument with a Cu Kα (λ = 1.5418 Å) X-ray source. Operating conditions were 30 kV, 15 mA in the 10°–80° 2Θ range, with a scan speed of 1.0° min^–1^.

### 2.4 Paired Electrolysis Measurements

**Scheme 1:**
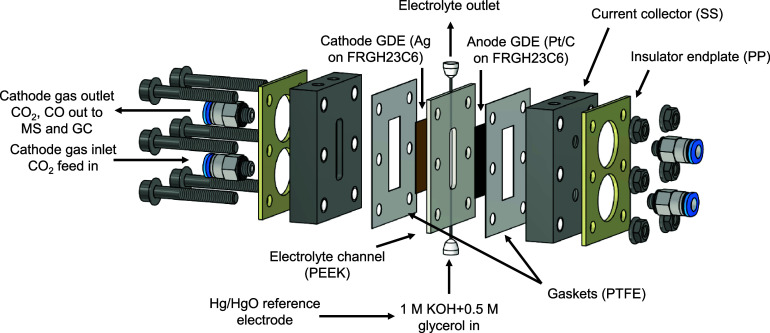
Schematic Exploded View of the Membraneless Microfluidic Electrolyzer Cell Employed in This Study SS = stainless steel, PP = polypropylene, PEEK = poletheretherketone, PTFE = polytetrafluoroethylene, GDE = gas diffusion electrode.

Continuous-flow electrolysis was carried out in a membraneless microfluidic electrolyzer cell in a three-electrode configuration (Scheme 1 and Scheme S1). The cell design was inspired by the one used by the Kenis group,^48^ and all cell components were constructed in-house. M5 threaded inlet and outlet ports were formed on both current collectors for gas transport. These channels were closed by blanking plugs on the anode current collector. The two stainless steel (SS) current collectors (1.4571, 316-Ti) sandwich a 2 mm thick flow channel (2 × 0.5 cm sized cavity, 1 mm holes drilled on both sides to ensure the transport of the electrolyte) made from PEEK. Three mm deep, 2 cm × 0.5 cm cavities were formed on each current collector to act as gas-flow channels. The sealing around the anode and cathode GDEs was established by using precut PTFE separators (200 and 100 μm thickness on the cathode and anode side, respectively). The electrolyte solution was flown through the cell using a peristaltic pump (Ismatec), applying a 5 cm^3^ cm^–2^ min^–1^ flow rate. One M KOH and 1 M KOH + glycerol in between 0.1 and 2 M concentration were used as electrolyte solutions. All measurements were carried out in the single pass mode. The electrolyte reservoir was constantly purged with Ar to avoid the penetration of O_2_ in the electrolyzer cell. CO_2_ was fed to the cathode GDE in a flow-by mode with a rate of approximately 12 cm^3^ min^–1^. The gas flow rate was controlled with a mass flow controller (MFC, Bronkhorst, EL-Flow Select F-201CV).

**Figure 1:**
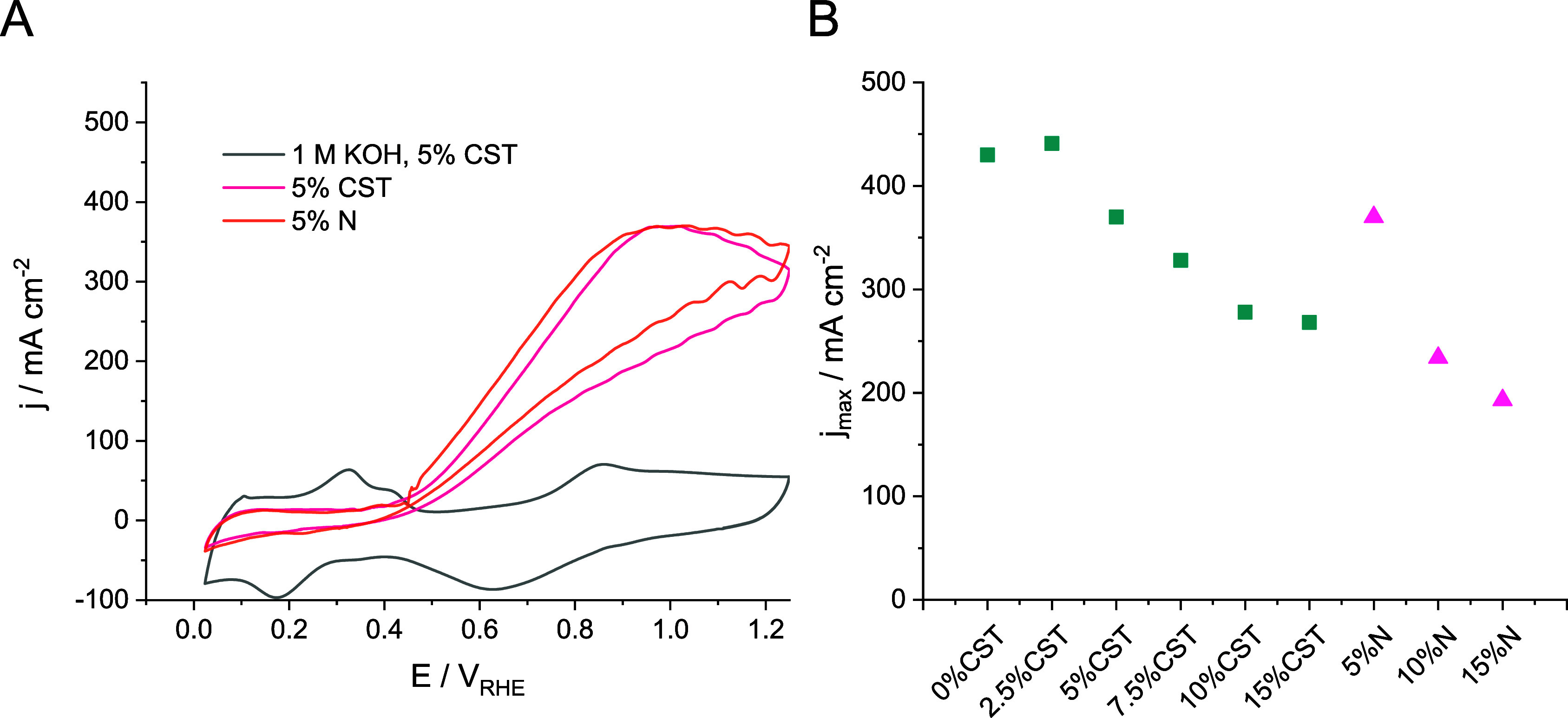
(A) Cyclic voltammograms recorded for the Pt/C samples prepared with 5 wt % ionomer content applying a 100 mV s^–1^ scan rate. CVs were collected in a membraneless microfluidic flow electrolyzer cell applying a 5 cm^3^ cm^–2^ min^–1^ electrolyte and 12 cm^3^ cm^–2^ min^–1^ CO_2_ flow rate. CST = Capstone ST-110, *N* = Nafion. Ten cycles were recorded in between 0 and +1.25 V_RHE_. Only the final cycle is presented in the figure. (B) Peak GOR current densities derived from the CVs presented in panel A and in Figures S5A,B and S6A, plotted as a function of the type and amount (in wt %) of ionomer in the catalyst layer.

Electrochemical measurements were performed using a Biologic VMP300 multichannel potentiostat/galvanostat. All data were collected in a three-electrode configuration, where a Hg/HgO/1 M KOH (Bioanalytical Systems Inc.) was employed as a reference electrode. It was connected to the inlet electrolyte stream via a T-connector. Gas-phase products formed at the cathode were monitored continuously using an SRS UGA200 atmospheric mass spectrometer and periodically with a Shimadzu Nexis GC-2030 gas chromatograph (operated with 6.0 He carrier gas). A barrier discharge ionization detector (BID) and an automatic 6-way valve injection system allowed the quantification of all formed gas-phase products with good precision. The flow rate of the outlet gas mixture was measured with an Agilent ADM flow meter, ensuring the proper calculations of Faradaic efficiency. Liquid-phase products formed at the anode were analyzed with NMR (Bruker AV-III-500-HD) and HPLC (Shimadzu Prominence LC-20AD liquid chromatograph). Calibration was always performed in electrolytes mimicking the environment of the real samples. Phenol and DMSO were applied as internal standards in the case of the NMR measurements (the liquid sample was diluted 10 times with ultrapure H_2_O and 50 μL of internal standard was mixed with 450 μL of diluted liquid sample).^48^ 1D ^1^H spectra were measured using a solvent presaturation method to suppress the water peak. The HPLC was equipped with a ReproGel H (9.0 μm, 300 × 8 mm) column and an SPD-M20A diode array detector. Five mM H_2_SO_4_ was used as the eluent with a flow rate of 0.5 cm^3^ min^–1^. The column temperature was set to 55 °C. In the case of both the NMR and HPLC measurements, each liquid sample was diluted (and neutralized) with 2 M H_2_SO_4_ (typically 250 μL to 1 cm^3^ collected liquid sample) to avoid the autocatalytic decomposition of glycerol oxidation products/intermediates.^49^ This sample (20 μL) was injected into the HPLC column.

## 3 Results and Discussion

### 3.1 Morphology and Composition of the Synthesized Catalyst Layers

Pt/C GDEs were prepared by spray-coating. Two different polymers, CST and Nafion, were used as catalyst additives/binders, and their amounts were varied between 0 and 15 wt % in the catalyst layers. As a first practical observation, the catalyst dispersion homogeneity was greatly improved by the addition of the ionomer. Insights on the catalyst structure were first gleaned by XRD (Figure S1A). Three diffraction peaks can be identified on the diffractogram centered at 39.7°, 46.0°, and 67.5° corresponding to the diffraction of the Pt (111), (200), and (220) lattice planes, respectively.^50^ The wide diffractions indicate that the size of the Pt nanoparticles is very small (a few nanometers, as estimated from the Scherrer equation). Morphology of the as-prepared GDEs was studied with SEM (Figure S2). The carbon-supported catalyst homogeneously covered the GDL surface. At higher magnification, even the Pt nanoparticles can be clearly observed as tiny, bright spots evenly distributed along the carbon substrate, as further supported by EDX mapping (Figure S3). The physical appearance of the ionomer cannot be identified even at the highest loading of 15 wt %. In addition to SEM, the morphology of the Pt/C catalyst was further scrutinized by TEM (Figure S1B), which demonstrated that the Pt nanoparticles are homogeneously distributed on the Ketjenblack carbon support. The diameter of most nanoparticles was between 2 and 4 nm (Figure S1C). XRD and SEM images were captured for the Ag and Ir black GDEs employed as a cathode and anode after electrolysis (see Figure S4 and the corresponding discussion).

### 3.2 Paired CO2RR and Glycerol Oxidation in a Flow Cell

Paired CO2RR/AOR electrolysis measurements were performed in a membraneless microfluidic electrolyzer cell (Scheme 1). The cell design (small cell size with the absolute minimum number of cell components with no membrane separation), together with the small geometric area of the GDEs (1 cm^2^ on both the anode and cathode sides) allows the rapid exploration of the effect of different experimental parameters. As the first step, 10 subsequent cyclic voltammograms (CVs) were recorded between 0 V_RHE_ and +1.25 V_RHE_ applying a 100 mV s^–1^ scan rate, in glycerol containing solutions; the final scans are presented in Figure 1A, Figure S5A, and Figure S6A. Note that the recorded curves are noisier than the ones typically measured in static H-cell setups due to the relatively high electrolyte flow rate of 5 cm^3^ cm^–2^ min^–1^. A control measurement was also performed for the Pt/C GDE containing a 5 wt % CST ionomer (gray curve); here, no glycerol was added to the KOH solution (Figure 1A). All oxidation/reduction peaks appeared on this latter CV curve are characteristic to polycrystalline Pt.^38^ As for the results recorded in the glycerol-containing solutions, the onset potential of GOR was ca. + 0.40 V_RHE_, which is in good agreement with reports in the literature.^32^ The GOR current density reaches its maximum at *E* ≈ +1.00 V_RHE_, after which it decreases rapidly. This can be attributed to the (partial) oxidation of the Pt surface along with the possible irreversible adsorption of GOR intermediates/products (since data were recorded under continuous electrolyte/CO_2_ flow, no mass transport limitation is present from the electrolyte side).

The achievable maximum current density showed a very strong dependence on the ionomer content of the catalyst layers (Figure 1B). The highest AOR current density was measured when the catalyst layer contained 2.5 wt % CST (*j* = 442 mA cm^–2^). If the ionomer content exceeded 5 wt %, the current density decreased notably. The reason behind this is the blocking of the accessible active sites by the increasing amount of ionomer in the catalyst layer. A similar measurement was performed, but replacing CST with Nafion (5, 10, and 15 wt %, Figure S6A). CVs recorded for the 5 wt % ionomer-containing GDEs run together (Figure 1A). However, when the Nafion content was further increased (Figure S6 and Figure 1B), notably smaller current densities were recorded compared to the samples prepared with identical CST amount (Figure S6 and Figure 1B), calling attention to the possible effect of the catalyst binder. After the potentiodynamic measurements, the GOR was studied by performing potentiostatic electrolysis between +0.88 V_RHE_ and +1.13 V_RHE_. The results are presented and discussed in Figures S5B and S6B. In brief, trends in terms of the achievable activity are identical to the ones observed in the case of the CVs.

Interestingly, current densities measured for the 5 wt % Nafion-containing system were notably less stable compared to the CST-containing counterparts (Figure S6B) and showed a rapid decrease from *E* = +1.08 V_RHE_. The achievable current densities decreased for both ionomers when their amount in the catalyst layer was further increased, and instead of increasing with the potential, the current density decreased even during the given potentiostatic step. We speculate that this phenomenon is caused by the excess amount of ionomer blocking a considerable number of active sites.^51^ Therefore, the reaction rate cannot be increased further (because only a limited amount of glycerol can approach and adsorb at the catalyst surface) with the increase of the driving force (i.e., the potential).

The current density increased with the applied electrolyte flow rate (Figure S7). Glycerol concentration also had a notable influence on the achievable current densities (Figure S8). At low concentrations (between 0.1 and 0.25 M), there is not enough glycerol close to the catalyst surface, limiting the reaction rate. The highest current densities were recorded when the glycerol content was between 0.25 and 0.5 M. When the amount of glycerol is even higher, the achievable current densities start to decrease, mainly attributed to the alteration of the electrolyte viscosity.^52^ In addition, high glycerol concentration had a strong influence on the selectivity of the cathode reaction because the presence of glycerol in the electrolyte solution alters its dielectric properties, which indirectly affects the wetting properties of both the anode and cathode GDE. This results in the flooding of the cathode GDE, shifting the selectivity toward HER (Figure S8C).

**Figure 2:**
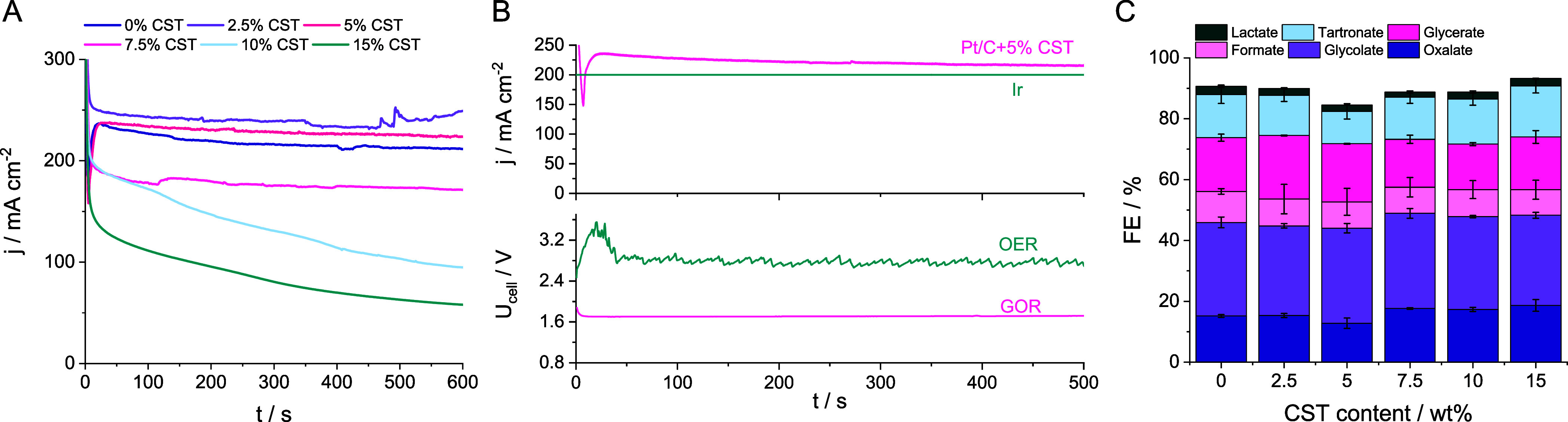
(A) Potentiostatic electrolysis at +1.03 V_RHE_ recorded for the Pt/C catalyst layers of varying ionomer content. (B) Typical cell voltage in a CO_2_ electrolyzer when OER is performed at the Ir anode catalyst, compared to an electrolyzer in which the OER is replaced with GOR. Conditions in the OER case were: *j* = 200 mA cm^–2^, 1 M KOH, 1 cm^3^ cm^–2^ min^–1^ electrolyte flow rate. Conditions in the GOR case are identical to the measurement presented in panel A: the electrolyte was 1 M KOH + 0.5 M glycerol flowed through the cell in a single pass mode. The electrolyte flow rate was set to 5 cm^3^ cm^–2^ min^–1^, while the CO_2_ flow rate was maintained at 12 cm^3^ cm^–2^ min^–1^. (C) Glycerol oxidation product distribution as a function of the CST ionomer content in the Pt/C catalyst layer. Samples were taken during the potentiostatic holds presented in panel A. Error bars were calculated for three measurements, each performed using fresh cathode and anode catalyst layers. All measurements were performed in a membraneless microfluidic flow electrolyzer cell.

Finally, to analyze the formed products at the cathode/anode, the potential was held at +1.03 V_RHE_ for 10 min (Figure 2A). At the cathode, the Faradaic efficiency (FE) of CO formation always exceeded 95% (Figure S9), as quantified by GC-BID measurements. The remaining charge was consumed by H_2_ formation. Similar ratios were seen on the MS data, where the partial pressure of products was followed; moreover, the CO partial pressure was stable throughout the 10 min long experiments. These observations show that the presence of 0.5 M glycerol in the electrolyte did not alter the stability and selectivity of the Ag cathode catalyst layer within the time frame of the measurement.

The highest stable current densities were recorded for Pt/C+2.5 wt % CST and 5 wt % CST, while the current density measured for the ionomer-free sample slowly but steadily decreased over the course of the 10 min long experiment. A grayish coloration of the electrolyte solution was observed in the case of the 0 and 2.5 wt % CST-containing samples, suggesting the slow mechanical decomposition of the catalyst layer. All further experiments were therefore performed with the 5 wt % CST-containing electrodes. For these, the cell voltage was 1.7 V (Figure 2B), in contrast to U_cell_ ≈ 2.7 V, which was measured when OER was performed at the anode of the CO_2_ electrolyzer at almost identical total current density. This proves that replacing the OER at the anode of the electrolyzer indeed lowers the overall cell voltage, and in turn, it can lower the energy needs of the electrolysis process. By optimizing the catalyst layer composition and operating conditions, the electrolyzer cell can be run at high current densities at approximately 1 V lower cell voltage!

Six C1–C3 liquid GOR products were identified (Figure 2C), namely: C3 products—glycerate, lactate, and tartronate; C2 products—glycolate and oxalate; and C1 product—formate. There is no clear trend regarding the influence of the CST content on the GOR selectivity, and the variation of each products’ share in the mix remained within the limits of experimental error. Glycerate, tartronate, oxalate, and glycolate production accounts for the majority of the passed charge. The type of ionomer also had almost no effect on the product distribution at low ionomer content (see Figure S6C,D and the corresponding discussion). The electrolyte flow rate influences the GOR product distribution, especially in terms of the amount of formate and glycolate (Figure S7B).

Unfortunately, exact selectivity data (providing FEs) are hard to come by in the literature, but our products and FE values are in the regime of what others reported using Pt as the catalyst.^7,24,25^ There are three reasons behind the total anodic FE falling below 100% (10–20% of the passed charge cannot be accounted for considering only the above products): (1.) C–C cleavage occurs in a Cannizzaro reaction (due to the highly alkaline environment), where it is hard to assign the exact number of consumed electrons, (2.) GOR intermediates can further decompose autocatalytically due to the strongly alkaline medium despite our efforts to freeze the reaction (adding 2 M H_2_SO_4_ to the liquid sample making it neutral/slightly acidic immediately after taking it). Dihydroxyacetone and glyceraldehyde were identified in some cases as products, but their amounts were not quantifyable.^49^ Finally, (3.) part of the glycerol was completely oxidized to CO_2_ (dissolving as CO_3_^2–^ in the alkaline solution), which is not detectable with the employed analytics under CO2RR conditions. We proved the formation of CO_2_ in a control experiment, and therefore, we strongly believe that majority of the missing FE is related to the complete oxidation of glycerol (Figure S10 and the corresponding discussion). Scheme S2 summarizes GOR pathways based on all detected (and quantified products).

### 3.3 Long-Term Stability

**Figure 3:**
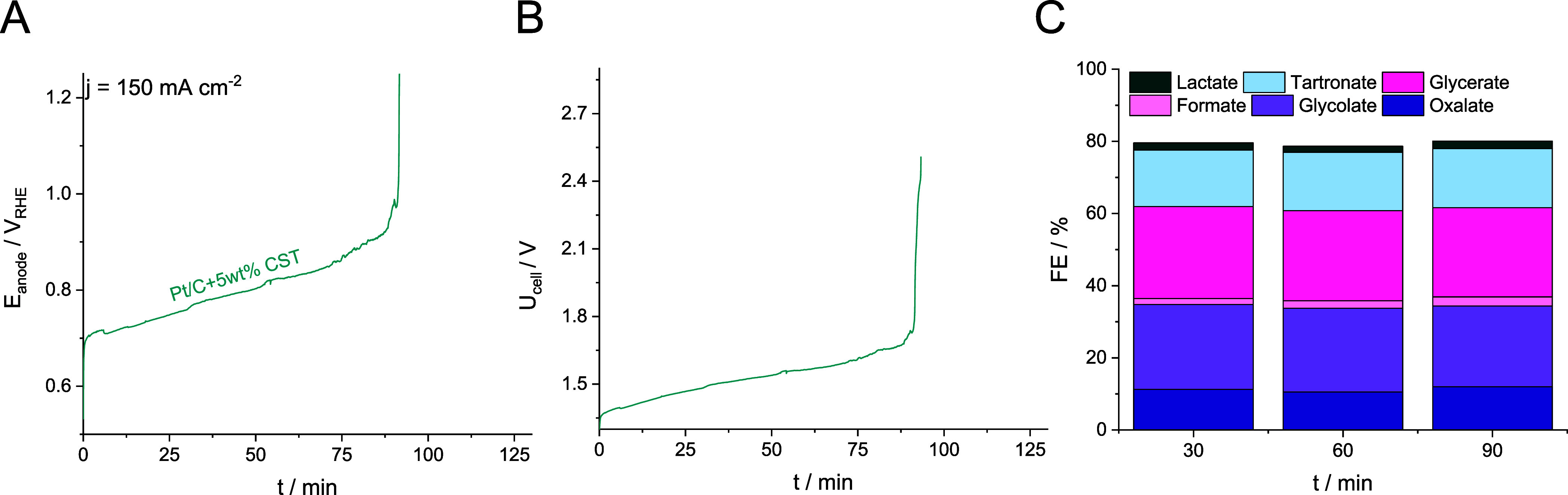
(A) Anode potential and (B) cell voltage during the measurement at *j* = 150 mA cm^–2^. The CO_2_ flow rate was maintained at 12 cm^3^ cm^–2^ min^–1^, while the 1 M KOH + 0.5 M glycerol electrolyte flow rate was set to 5 cm^3^ cm^–2^ min^–1^. All measurements were performed in a membraneless microfluidic flow electrolyzer cell. (C) GOR product distribution during the measurement shown in (A)–(B).

The long-term operation of the CO2RR/GOR electrolyzer cell was first evaluated in galvanostatic experiments at *j* = 150 mA cm^–2^, while the cell voltage and anode potential were simultaneously monitored (Figure 3A,B). All measurements were conducted with a single pass of the 1 M KOH + 0.5 M glycerol electrolyte solution, thus preventing the interaction (adsorption/reoxidation, etc.) of the GOR products or any dissolved metal species with the cathode or anode catalyst layer. The anode potential, in conjunction with the cell voltage, monotonously increased during electrolysis up to a point (typically around 90 min) when both values suddenly jumped to the ones typically measured when the OER is performed at the anode. The GOR selectivity (Figure 3C) also remained stable until approximately 90 min. Oxalate (≈11–12% FE), glycolate (≈22–23% FE), formate (≈<2% FE), glycerate (≈23–25% FE), tartronate (≈15% FE), and lactate (≈2% FE) formed as products. As the anode potential increases, the GOR selectivity slightly shifts toward the production of formate, along with the decrease of the formed glycerate and glycolate amount. The sudden increase in cell voltage when using Pt as the anode catalyst was already observed in the literature, and it was ascribed to the formation of a compact PtO_*x*_ overlayer on top of Pt.^25^ PtO_*x*_ is inactive toward GOR, hence the sudden increase in the cell voltage. The mechanism of catalyst deactivation under galvanostatic experiments is presumably the following: at 150 mA cm^–2^, GOR progresses on Pt at potentials under which the Pt surface starts to oxidize. This manifests as oxide domains at the beginning rather than a continuous overlayer, decreasing the number of available active sites. To maintain the applied current density, the necessary anode potential (and the cell voltage) starts to increase up to a point when the Pt surface is fully passivated by the formed oxide. As a result, the anode potential jumps to values at which the OER can be conducted. Based on our results, it seems that galvanostatic electrochemical protocols applying high current densities cannot be maintained for long using Pt as the anode catalyst. We hypothesized that the rate of oxide formation could be better controlled if the anode potential is controlled instead of the applied current density. It might mean that the measured current density changes along the measurements, but the operation of the coupled CO2RR/GOR electrolyzer can be maintained longer.

**Figure 4:**
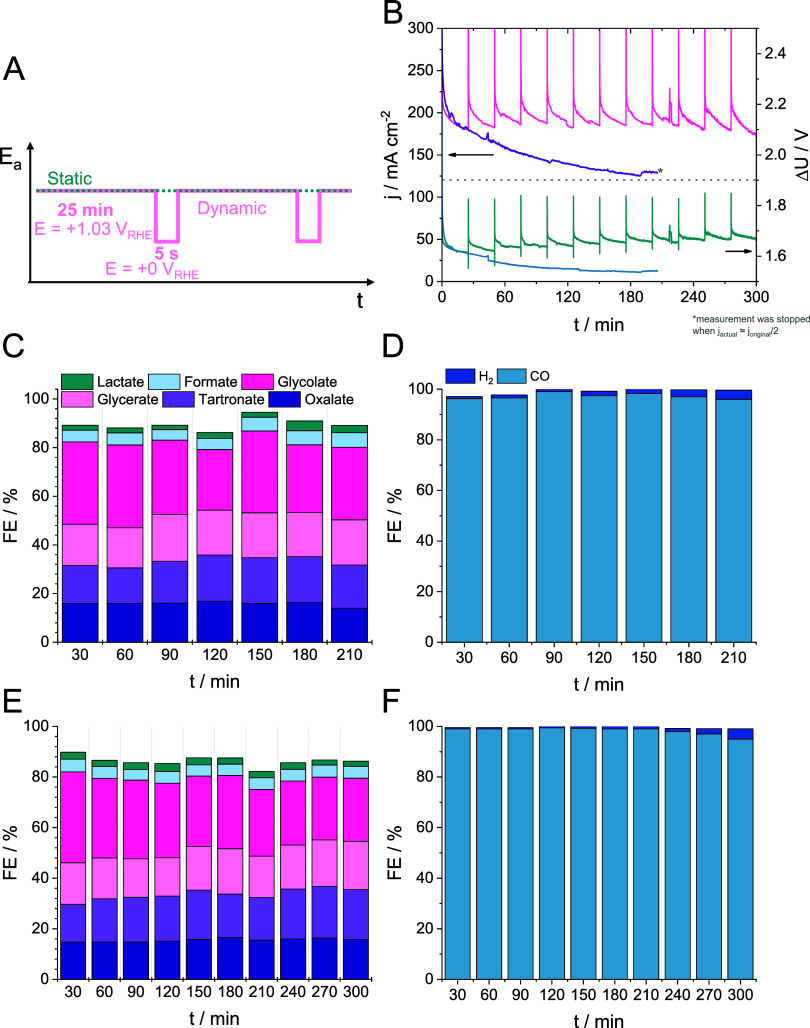
(A) Differences between the static and dynamic electrochemical protocols. (B) Current density vs time and the resulting cell voltage measured during long-term coupled CO2RR/GOR by applying static (teal curve) and dynamic (pink curve) chronoamperometric measurements according to panel A. All measurements were performed in a membraneless microfluidic flow electrolyzer cell in 1 M KOH + 0.5 M glycerol electrolyte solution, which was not recirculated during the measurement. The electrolyte flow rate was maintained at 5 cm^3^ cm^–2^ min^–1^, while the CO_2_ flow rate was set to 12 cm^3^ cm^–2^ min^–1^. GOR (C) and CO2RR (D) product distributions measured during the static electrochemical protocol. GOR (E) and CO2RR (F) product distributions measured during the dynamic electrochemical protocol.

As a next step, the anode potential was set to *E* = +1.03 V_RHE_ while monitoring the current density (Figure 4A,B (purple trace)). The initial high current density (above 200 mA cm^–2^) decreased continuously from the beginning of the experiment, reaching 50% of its initial value after about 3 h. The change in the current density is accompanied by the decrease in cell voltage (teal curve in Figure 4A). The selectivity at both the anode and cathode side remains stable throughout the measurement (Figure 4C,D) and matches the values obtained during the galvanostatic measurement presented previously. At the cathode, there is a slight increase in the amount of formed H_2_, due to the gradual wetting of the cathode GDE, but 96% of the passed charge is still consumed by CO formation after 180 min of the operation. Overall, CO2RR/GOR can be conducted considerably longer when the anode potential is controlled instead of the current density because of the slower PtO_*x*_ formation rate. The oxidation of the catalyst surface, however, cannot be fully mitigated under high current density GOR conditions, which leads to its gradual deactivation (see Figure S11 and the corresponding discussion).

### 3.4 Dynamic Operation to Reactivate the Anode Catalyst

The gradual deactivation of the Pt/C GOR catalyst experienced during the potentiostatic measurements could be reversed by changing the static electrochemical protocol to a dynamic one. The differences between the static and dynamic electrochemical protocols applied here are summarized in Figure 4A. A reductive potential is applied after conducting the potentiostatic measurement (*E* = +1.03 V_RHE_) for 25 min, allowing the catalyst surface to be reduced. The potential was set to *E* = +0 V_RHE_ for only 5 s, which allows the reduction of the previously formed surface passivating PtO_*x*_ and the restoration of the catalyst surface. Notably, PtO_*x*_ reduction is always accompanied by the dissolution of a small amount of Pt.^37^ Thus, the oxide reduction/metal dissolution process is responsible for the renewal of the catalyst surface.

As shown in Figure 4A,B (and in Figure S12), the dynamic electrochemical protocol allows the activity of the anode catalyst layer to be preserved, and the electrolyzer cell can be operated at high current densities (around 200 mA cm^–2^), for 5 h continuously. The dynamic operation alters the GOR selectivity only marginally (Figure 4C,E): seemingly a larger fraction of the charge is consumed by glycerate, tartronate, and glycolate formation. The product distribution at the cathode is unaffected by the mode of operation (Figure 4D,F).

The post long-term electrolysis structure of the anode catalyst layers was first scrutinized with XRD (Figure S13). No change in diffraction intensity and diffraction position can be observed, as compared with the fresh samples (Figure S1A). The TEM images captured for the samples after the long-term measurement (Figure S14A–C) are also very similar to those recorded for the pristine sample (Figure S1B); there are no striking differences in the particle size and Pt distribution on the carbon matrix. To strengthen this statement further, the particle sizes of 150 nanoparticles were measured (Figure S14D). The number of bigger NPs decreased when the “static” protocol was performed, along with the increase in the number of smaller (1–3 nm) NPs. Contrastingly, the amount of the smaller NPs (between 1 and 2.5 nm diameter) notably decreased in parallel with the increase in the share of NPs with 2.5–3.5 nm diameter when the “dynamic” protocol was performed. This phenomenon is well-documented for Pt/C fuel cell catalysts that were tested under accelerated stress test protocols (typically fast potential cycling in a relatively wide potential window). The reason behind it is the agglomeration/Ostwald ripening of the Pt NPs due to the periodic switching in between the oxidative (here, +1.03 V_RHE_) and reductive (here 0 V_RHE_) potentials.^53−55^ Ostwald ripening is an indirect process, which is linked to Pt dissolution: smaller Pt NPs dissolve in the electrolyte, and the dissolved metal ions redeposit on the surface of the larger NPs thanks to the switching in the applied potential. Agglomeration can also occur through the migration of NPs when they are in the proximity of each other. The corrosion of the carbon support can facilitate this process—as the support shrinks Pt NPs get in contact with each other and agglomerate.^53^

## Conclusions and Outlook

In this contribution, OER, the typically applied anode process of CO_2_ electrolyzer cells was replaced with the electrochemical oxidation of glycerol. We demonstrated that, by optimizing the anode catalyst layer composition (quality and quantity of the used ionomer), the necessary cell voltage can be decreased by almost 1 V compared to a scenario when OER is conducted at the anode, in both cases operating at ca. 200 mA cm^–2^ current density, with stable selectivity for 5 h. To achieve this result, the following factors were of prime importance:

- Ionomer content. It has to be high enough to ensure the mechanical stability of the catalyst layer, but too much could block the active sites of the anode catalyst.
- Potential control instead of current control during the long-term electrochemical protocols. The rate of PtO_*x*_ formation can be tamed by controlling the anode potential instead of the current density extending the lifetime of the anode catalyst.
- Dynamic instead of static electrochemical protocols. These can alleviate issues stemming from the passivation of the catalyst surface by periodically reducing it.

Our future research efforts are dedicated to better understanding the factors causing the deactivation of the anode catalyst layer (the catalyst oxidation state in the presence and absence of glycerol, adsorbed glycerol oxidation intermediates) to mitigate Pt corrosion during the dynamic long-term measurements. The goal is to design electrochemical protocols that allow one to conduct coupled CO2RR/GOR measurements in a zero-gap cell configuration, providing a scalable platform. An additional benefit of the GOR in the zero-gap cell configuration is that the only gas-phase product (if any) at the anode side is CO_2_ that can be directly recirculated to the cathode side after condensing the alcohol and water content. This eliminates the CO_2_/O_2_ separation step that would be necessary if OER was conducted as the anode reaction, reducing the CO_2_ loss.^56^

## Supporting Information Available

(Schemes S1 and S2 and Figures S1–S14) Structure and photograph of the membraneless microfluidic electrolyzer cell employed in this study; additional material characterization data: SEM-EDX and TEM measurements, cyclic voltammograms, chronopotentiometric holds recorded for the anode catalyst layer prepared with different amounts of CST and Nafion ionomers, along with additional selectivity data both for the cathode and anode reaction; the effect of the electrolyte flow rate and glycerol concentration on the GOR activity; identification of CO_2_ as a GOR product by *in situ* mass spectrometry; mechanism of the GOR; additional data regarding the long-term measurements recorded during the dynamic operation of the paired electrolyzer cell; and postlong-term measurement characterization data of the anode catalyst layers performed by XRD and TEM (PDF)
