# Supplementary material for: Stable Operation of Paired CO2 Reduction/Glycerol Oxidation at High Current Density
Source: ACS Catal. 2024 Apr 13;14(9):6503–12. doi: 10.1021/acscatal.3c05952 (PMC11075010; doi:10.1021/acscatal.3c05952)
Supplement: Supplementary file 1 — cs3c05952_si_001.pdf [file cs3c05952_si_001.pdf]

Supporting Information

Stable Operation of Paired CO_2 Reduction/Glycerol Oxidation at High Current Density

Attila Kormányos,[1,] Adrienn Szirmai,[1] Balázs Endrődi,[1] Csaba Janáky[1,*]*

[1]Department of Physical Chemistry and Materials Science, University of Szeged, Aradi sq. 1, Szeged, 6720, Hungary

*Corresponding author, e-mail: kormanyos.attila@szte.hu, janaky@chem.u-szeged.hu

Table of Contents

Membraneless microfluidic flow cell S3

Morphology and Structure of the As-Prepared Catalyst Layers S4-S6

Ionomer-dependent GOR activity S7-S8

The effect of electrolyte flow rate on the GOR activity S9

Glycerol concentration dependent GOR activity and selectivity S10

CO2RR product distribution S11

Identification of CO_2 as a GOR product S12

Long-term operation below the GOR peak potential S13

Mechanism of the glycerol oxidation reaction S14

Long-term electrolysis S15

Post-LTM characterization S16-S17

Membraneless microfluidic flow cell

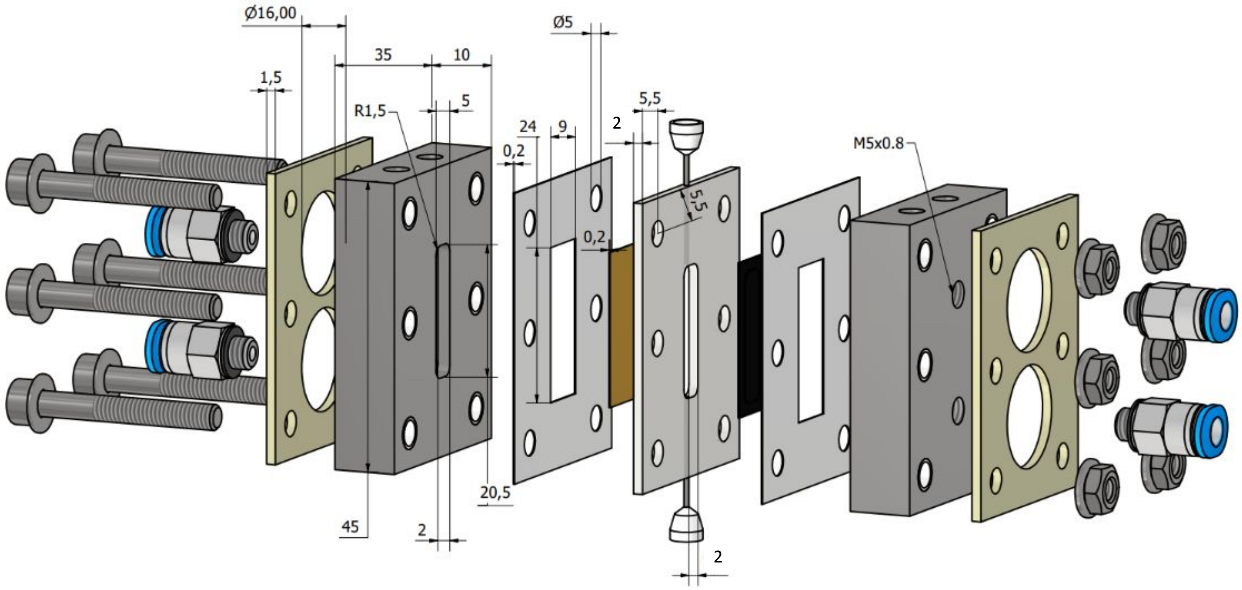

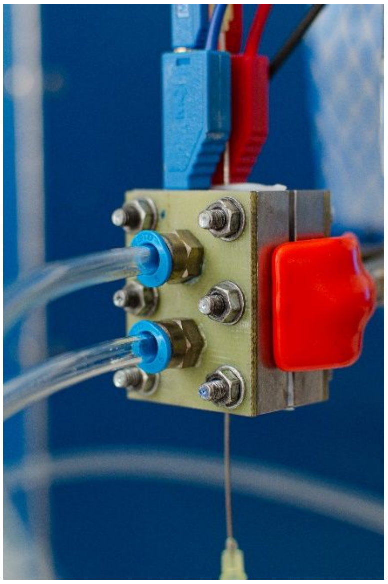

Scheme S1. Schematic exploded view of the membrane-less microfluidic flow electrolyzer cell employed in this study including the size of each cell component (top), and photograph of the assembled cell (bottom). All numbers on the scheme are in mm units.

Morphology and Structure of the As-Prepared Catalyst Layers

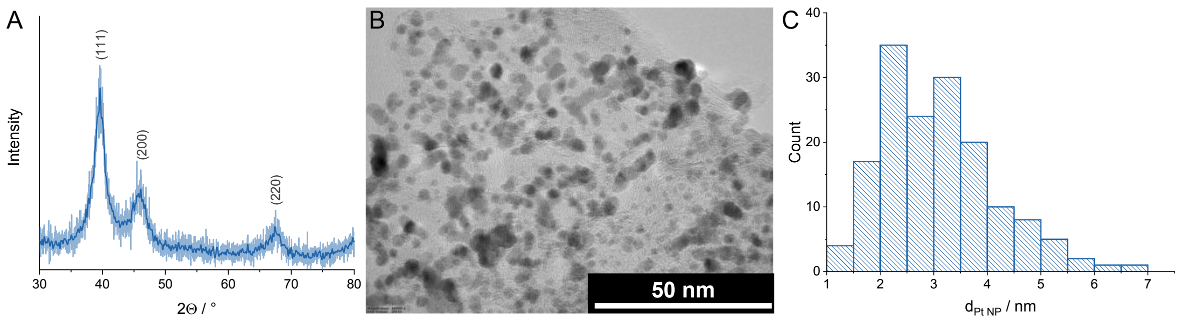

Figure S1. (A) XRD pattern measured for the as-prepared Pt/C catalyst layer. The obtained data was smoothed using an FFT filter (Points of window = 5). The original dataset is presented behind the smoothed curve with 50% transparency. (B) TEM image recorded for the Pt/C catalyst and (C) Pt nanoparticle size distribution calculated from the TEM data measuring the diameter of 150 nanoparticles from multiple images, recorded at different sites on the sample.

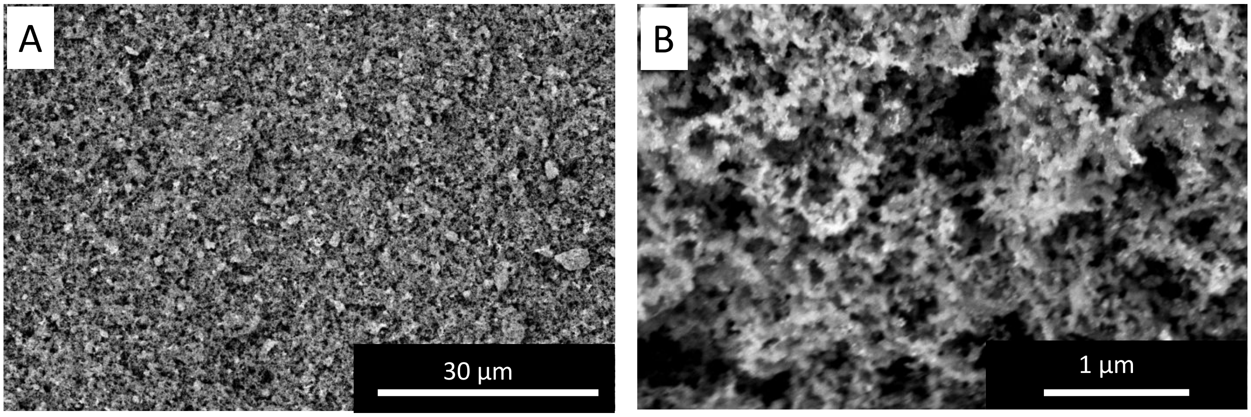

Figure S2. SEM images captured for the as-prepared Pt/C GDE+5 wt% CST with (A) 50000x and (B) 100000x magnifications.

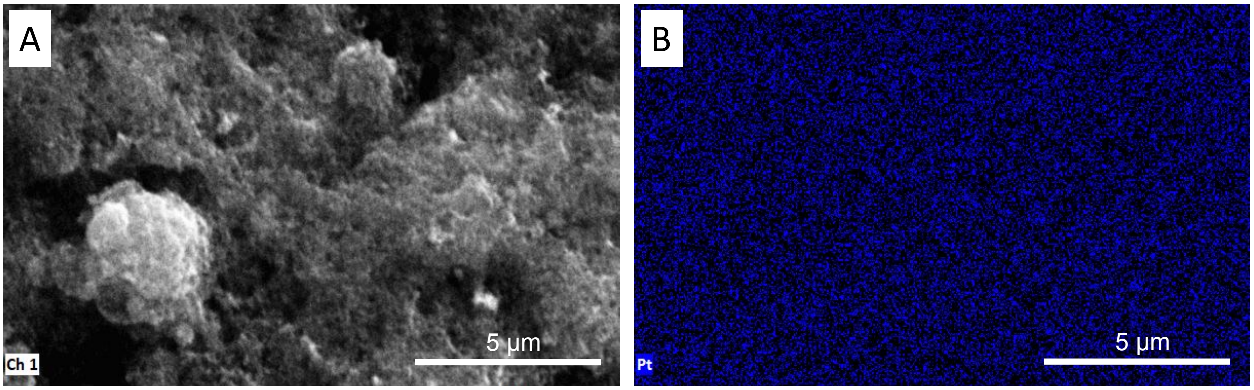

Figure S3. SEM image (A) captured for the as-prepared Pt%C GDE+5wt% CST and Pt distribution (B) in the nanocarbon support scrutinized with SEM-EDX.

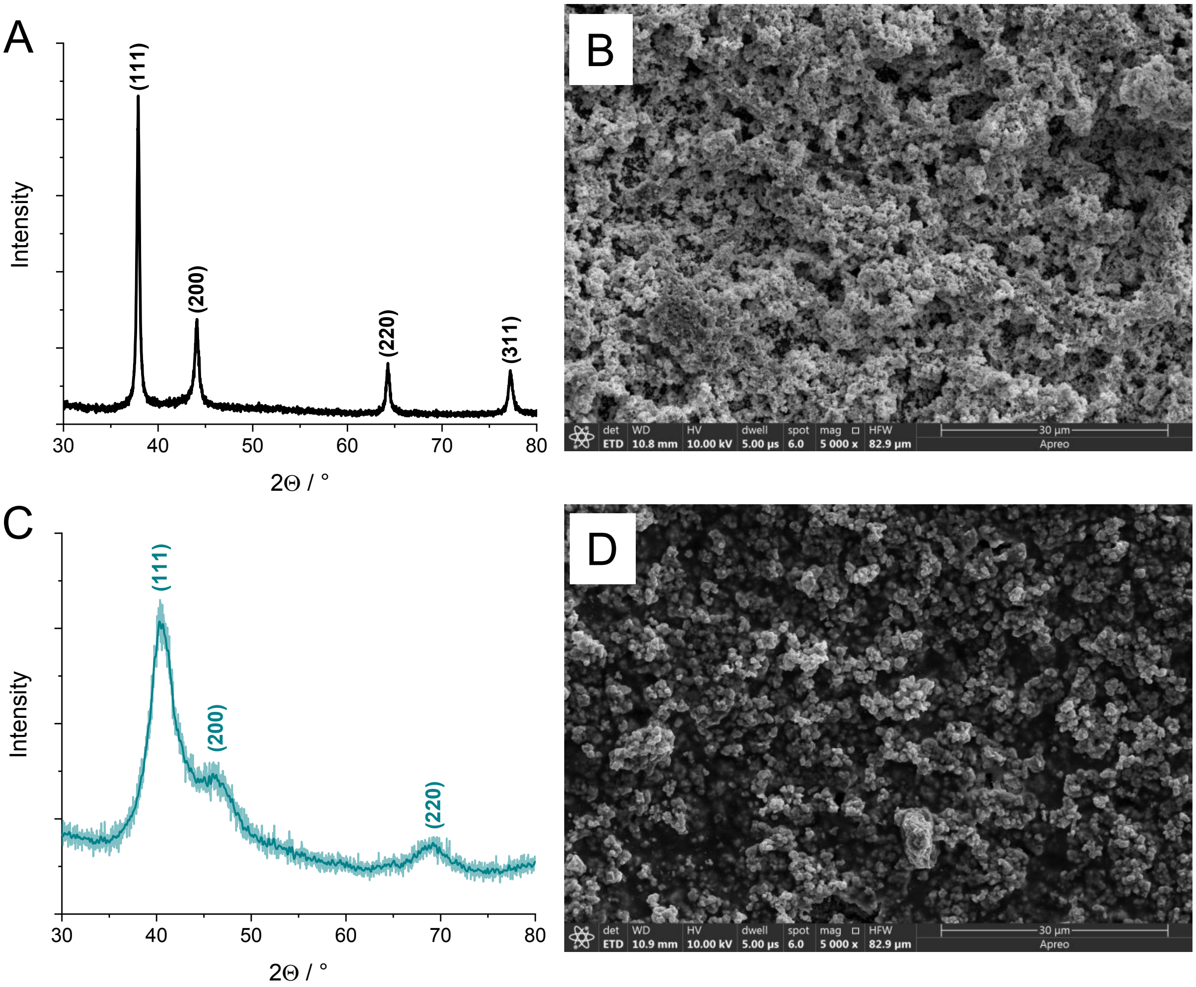

Figure S4. XRD recorded for the Ag NPs-coated cathode (A) and Ir black-coated anode (C) GDE. SEM images captured from the Ag (d<100 nm)+10 wt% CST ionomer-coated GDE (B) and from the

Ir black+15 wt% Nafion-coated GDE (D). The diffractogram recorded for the Ir sample was smoothed using an FFT filter (points of window=5), but the original dataset is presented behind the smoothed curve with 50% transparency.

Ag NPs:

Four diffractions can be identified in the XRD recorded for the Ag nanoparticles corresponding to the diffraction of the Ag (111), (200), (220) and (311) lattice planes, respectively.[1] According to the SEM image presented in Figure S3B, Ag NPs are evenly distributed along the Freudenberg H23C6 carbon paper gas diffusion layer substrate. The presence of 10 wt% CST can't be unequivocally identified.

Ir black:

Three diffractions can be observed in the XRD recorded for the Ir black NPs, which correspond to the (111), (200), (220) lattice planes of Ir, characteristic to an fcc crystal structure.[2] Similar to the Ag GDE, the Ir black NPs formed a homogeneous coating on the surface of the GDL. The presence of the 15 wt% Nafion ionomer can be spotted on the SEM image (Figure S3D) manifested as an amorphous matrix around the NPs.

Ionomer-dependent GOR activity

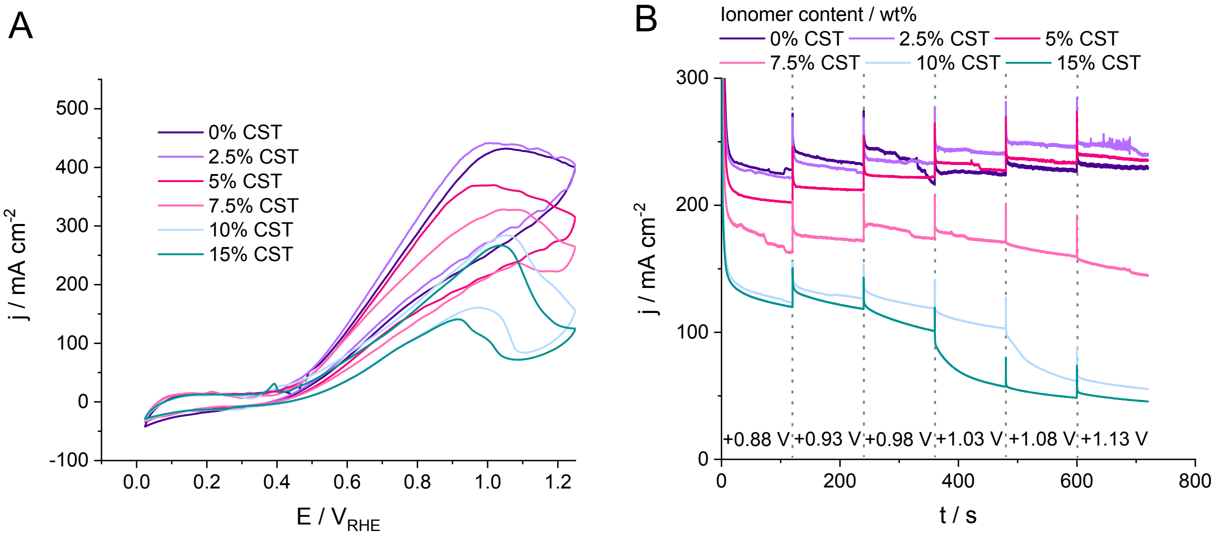

Figure S5. (A) Cyclic voltammograms recorded for the Pt/C samples prepared with different ionomer content applying 100 mV s^{-1} scan rate. CVs were collected in the membraneless microfluidic electrolyzer cell. CST = Capstone ST-110. 10 cycles were recorded in between 0 V_{RHE} to +1.25 V_{RHE}. (B) Chronoamperometric curves recorded at different potentials between +0.88 V_{RHE} and +1.13V_{RHE} (ΔE = 50 mV) recorded for the Pt/C samples prepared with varying the ionomer content. The duration of each hold was 2 minutes. All presented data was recorded in 1M KOH (+0.5 M glycerol) electrolyte solution, performing CO2RR at the cathode on a Ag-coated carbon GDL.

After the potentiodynamic experiments, the GOR activity of the Pt/C samples was studied by performing potentiostatic electrolysis between +0.88 V_{RHE} and +1.13 V_{RHE} with 50 mV increments (**Figure S5B**). The goal was to narrow this relatively wide potential window by finding the potentials at which GOR can be performed at the highest reaction rate with good stability. We note here that galvanostatic protocols are usually applied in the scientific community or the cell voltage is controlled rather than the anode potential. However, by controlling the anode potential, we can better monitor the performance fading of the catalyst that is most likely related to PtO_x formation. In terms of the trends, similar conclusions can be drawn as in the case of the CVs: highest current densities were measured if the ionomer content was equal or less than 5 wt%. The measured current density monotonously increased with the increasing potential reaching a maximum of 250 mA cm^{-2}, in the case of the Pt/C+2.5wt% CST sample at +1.08 V_{RHE}. High current densities were accompanied with considerable stability in the case of the 2.5 wt% and 5 wt% samples.

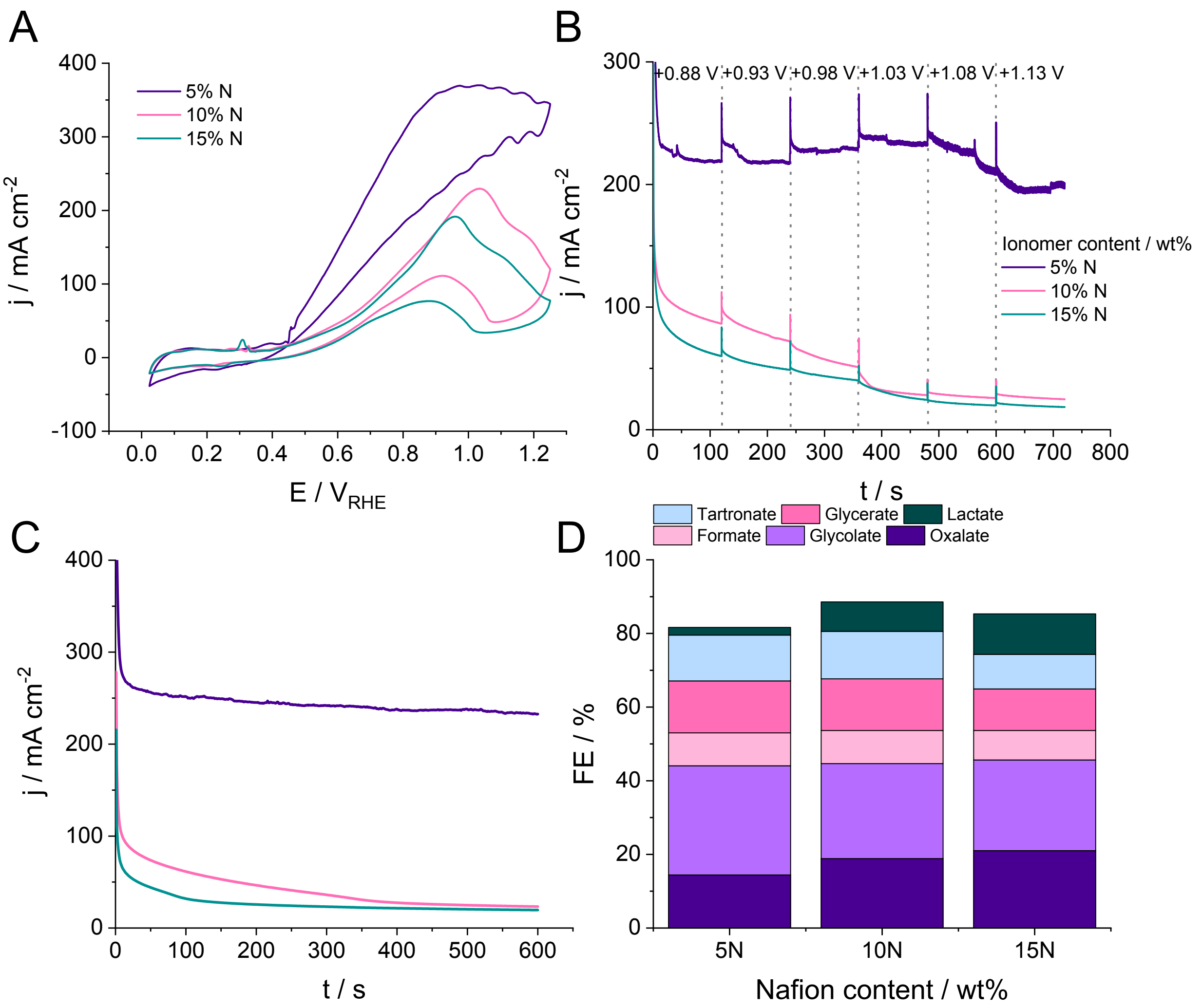

Figure S6. (A) Cyclic voltammograms recorded for the Pt/C samples prepared with different ionomer content applying 100 mV s^{-1} scan rate. N = Nafion. 10 cycles were recorded in between 0 V_{RHE} to +1.25 V_{RHE}. (B) Potentiostatic holds in between +0.88 V_{RHE} and +1.13V_{RHE} (ΔE = 50 mV) recorded for the Pt/C samples prepared with varying the ionomer content. The duration of each hold was 2 minutes. All presented data was recorded in 1M KOH (+0.5 M glycerol) electrolyte solution. (C) Potentiostatic hold at +1.03 V_{RHE} for 10 minutes recorded for the Pt/C catalyst layers prepared by varying the ionomer content. (D) Glycerol oxidation product distribution as the function of samples prepared with varying the ionomer content. Samples were taken during the potentiostatic holds presented in C. All measurements were performed in a membraneless microfluidic flow electrolyzer cell.

When the amount of Nafion was higher than 5 wt%, considerably higher fraction of the charge was consumed by lactate and oxalate formation. The measured current densities decreased to 25 mA cm $^{-2}$ and 19.5 mA cm^{-2} in the case of the 10 wt% and 15 wt% Nafion-containing catalysts, respectively, which might limit the comparability of our results.

The effect of electrolyte flow rate on the GOR activity

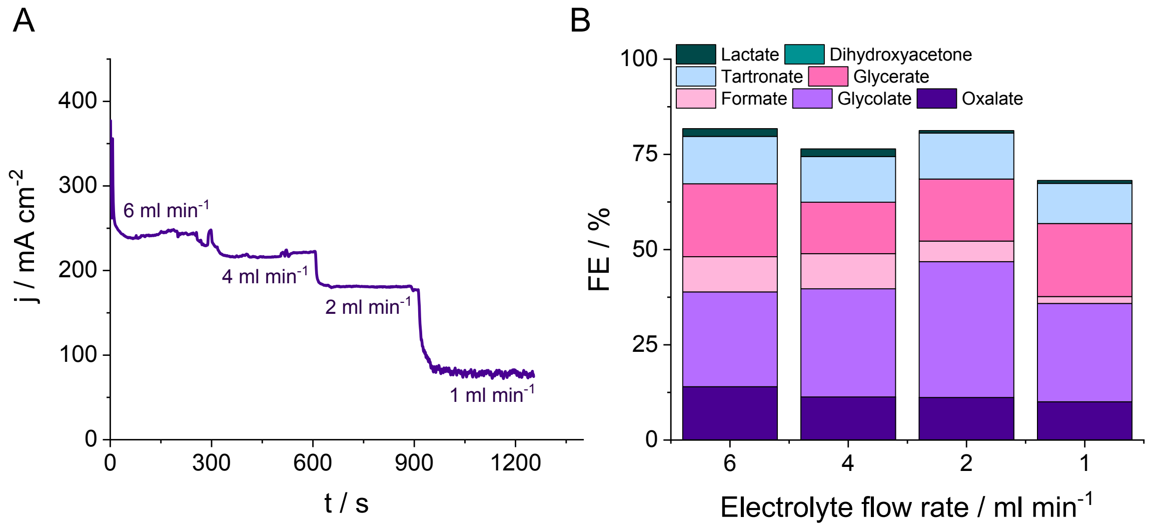

Figure S7. Potentiostatic measurements, depicting the influence of the applied electrolyte flow rate on the GOR activity (A) and on the GOR selectivity (B). The electrolyte flow rate was varied in between 1 cm^3 cm^{-2} min^{-1} and 6 cm^3 cm^{-2} min^{-1}. The CO_2 flow rate was maintained at 12 cm^3 cm^{-2} min^{-1}. Measurements were performed applying E = +1.03 V_{RHE} anode potential in 1 M KOH+0.5 M glycerol solution.

Glycerol concentration dependent GOR activity and selectivity

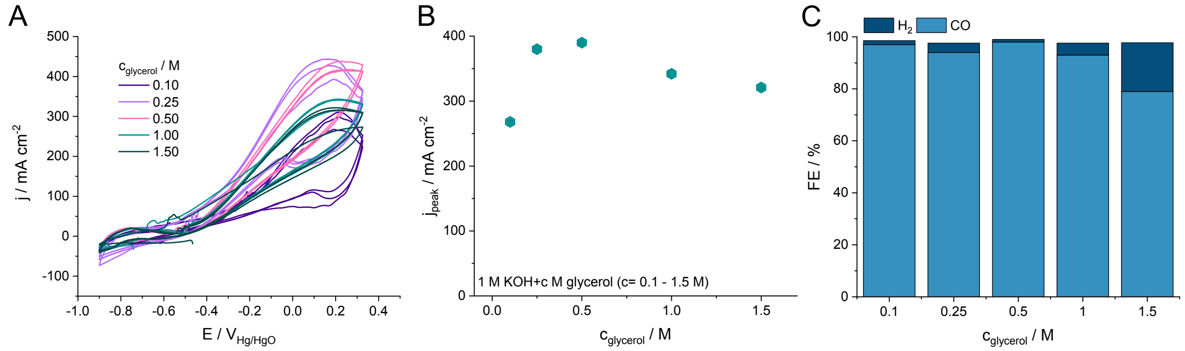

Figure S8. (A) Cyclic voltammograms recorded for the Pt/C samples prepared with the addition of 5 wt% CST ionomer, applying 100 mV s^{-1} scan rate. CVs were collected in the membrane-separated microfluidic flow electrolyzer cell. 10 cycles were recorded in between 0 V_{RHE} to +1.25 V_{RHE}. (B) Peak glycerol oxidation current densities derived from A. (C) Product distribution at the cathode measured by GC during a 10 min-long potentiostatic hold at E = +0.97V_{RHE}.

CO2RR product distribution

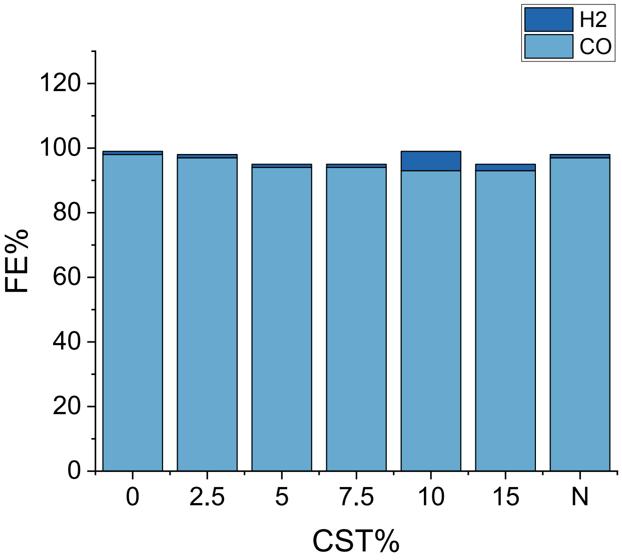

Figure S9. FE_{CO} and its dependence on the ionomer content of the anode GDE. CST = Caspstone ST-110, N = 5 wt% Nafion.

Identification of CO_2 as a GOR product

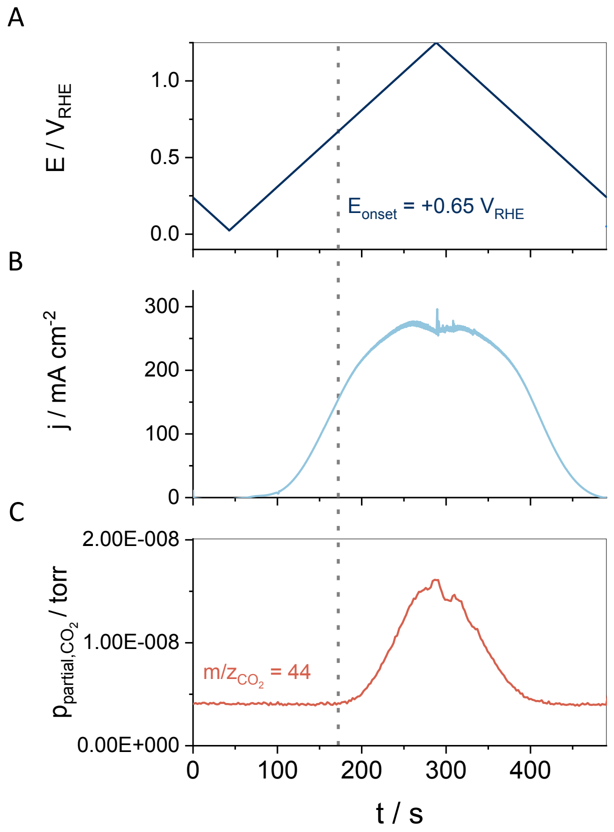

Figure S10. (A) Anode potential vs. time plot recorded for a CO2RR/GOR electrolyzer utilizing a Pt/C+5 wt% CST anode catalyst. The voltammogram was recorded applying 5 mV s^{-1} scan rate in between 0 V_{RHE} and 1.25 V_{RHE}. CO_2 was fed to the cathode side, while Ar was fed to the anode side of the cell with 12 cm^3 cm^{-2} min^{-1} flow rate. The purpose of this was to allow the transfer of the formed gas phase products towards the gas analyzer MS. The electrolyte flow rate was 5 cm^3 cm^{-2} min^{-1}. (B) The corresponding current density vs. t plot. (C) Partial pressure of the CO_2 formed at the anode during the electrochemical protocol presented in "A" and carried by the Ar flow to a residual gas analyzer MS, allowing the monitoring of the amount of formed products *in situ*. The delay between the electrolyzer cell and the MS has been corrected accordingly.

A constant flow of Ar was fed to the anode while the gas outlet was connected to the MS to monitor the composition of the gas stream in real-time, while no CO_2 was fed to the cathode (HER proceeded here). A CV in between 0 V_{RHE} and +1.25 V_{RHE} was recorded applying 5 mV s^{-1} sweep rate. A very small amount of CO_2 was detected from approximately +0.65 V_{RHE}, and its amount increased gradually until reaching the upper potential limit. This is an upper estimate for the onset potential of CO_2 formation, as the majority of CO_2 is immediately dissolved in the form of CO_3^{2-} and only a small amount can escape from the electrolyte solution (after saturating the locally formed carbonate buffer). Still, this experiment proves that CO_2 formation is unavoidable even at much lower potential than the GOR peak maximum. Scheme S2 summarizes GOR pathways based on all detected (and quantified products).

Long-term measurement below the peak potential of GOR

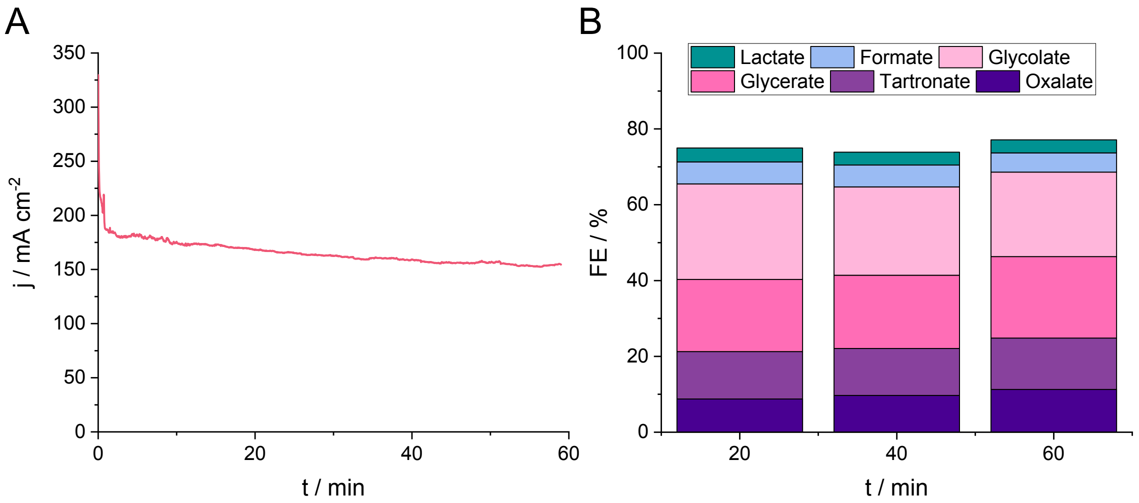

Figure S11. (A) Potentiostatic experiment performed using the Pt/C+5wt% CST anode catalyst layer applying E_{anode} = +0.88V_{RHE} potential. The measurement was performed in 1M KOH+0.5 M glycerol electrolyte solution. The electrolyte flow rate was maintained at 5 cm^3 min^{-1} cm^{-2}, while the CO_2 flow rate was set at 12 cm^3 min^{-1} cm^{-2}. (B) GOR product distribution during the long-term protocol presented in (A).

A potentiostatic electrochemical measurement was performed applying E_{anode} = +0.88 V_{RHE} potential. As shown in **Figure S11**, the current density immediately starts to decrease after the start of the measurement reaching around 152 mA cm^{-2} after 1 h of operation. The rapid decrease can be explained again by the surface passivation due to PtO_x formation. According to the CV presented in Figure 1A, the oxidation of the electrode surface starts well below +0.8 V_{RHE}. The further decrease of the anode potential, however, would result in significantly lower achievable current densities. Regarding the selectivity (Figure S11B), a bit higher amount of the passed charge was consumed by C2-C3 product formation.

Mechanism of the glycerol oxidation reaction

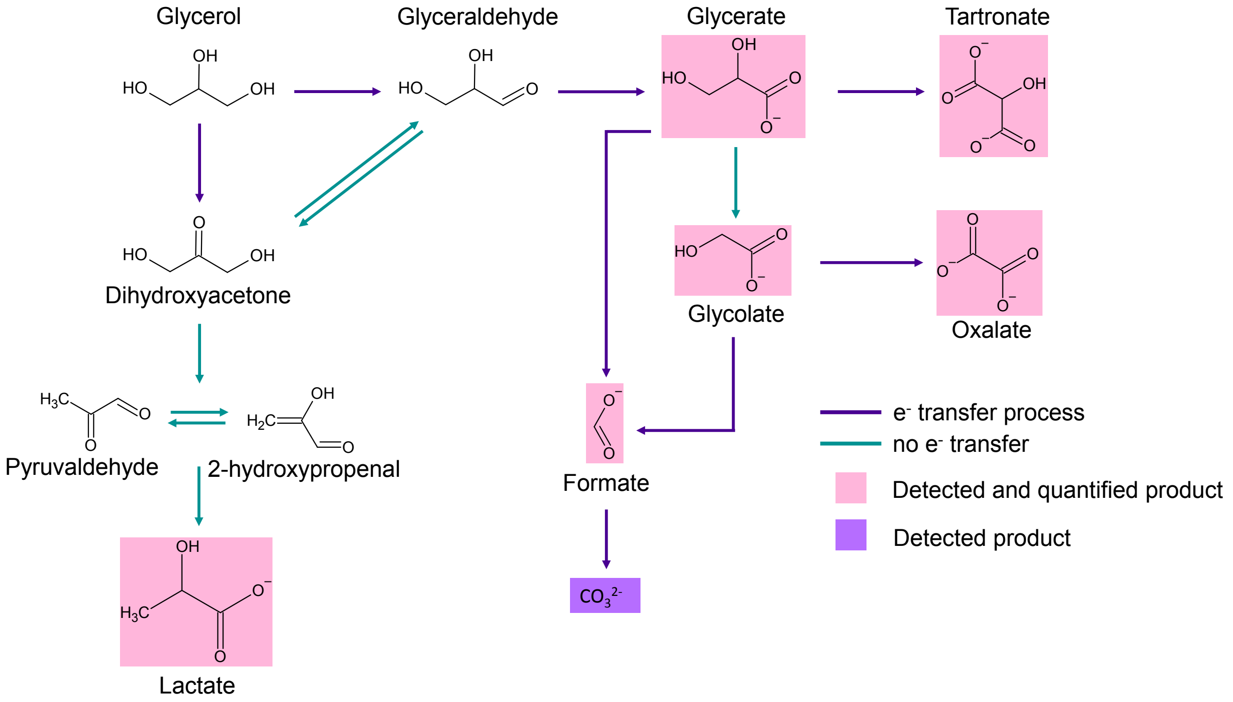

Scheme S2. Schematic summary of the various glycerol oxidation reaction pathways. Redox reactions (purple arrows) and non-redox reactions (teal arrows) are marked with different colors. All products that have been identified and quantified are framed by pink and the ones, which were only identified are marked with a purple frame.

Current density and cell voltage measured during the reductive pulse when performing the dynamic electrochemical protocol

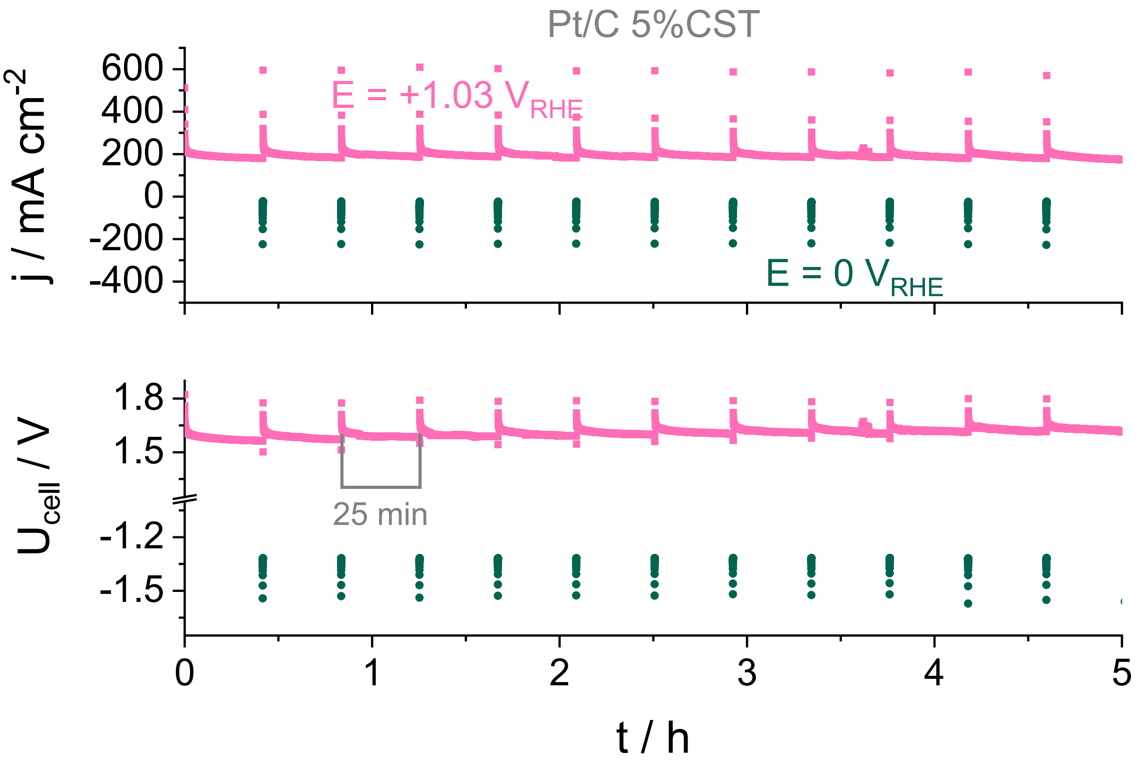

Figure S12. Current density vs. time (top), and the resulting cell voltage (bottom) measured during the dynamic long-term electrochemical protocol. The dynamic protocol consisted of 25 min-long potentiostatic holds at E = +0.97 V_{RHE} anode potential (pink curves), which were periodically interrupted by short potential pulses at E = 0.05 V_{RHE} (teal curves) lasting for 5s.

Post-Long Term Measurement Characterization

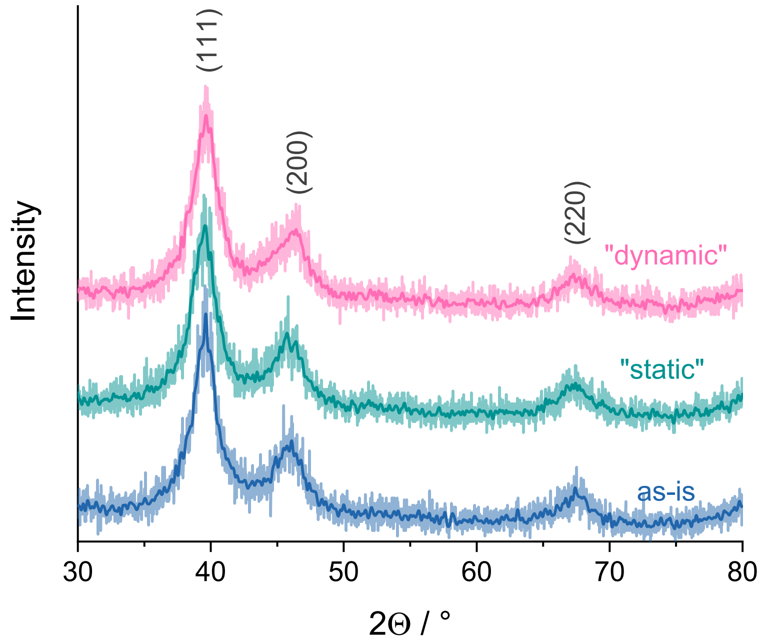

Figure S13. XRD data recorded for the pristine Pt/C+5wt% CST catalyst layer and for the catalyst layers after performing either the "static" or "dynamic" long-term electrochemical protocols.

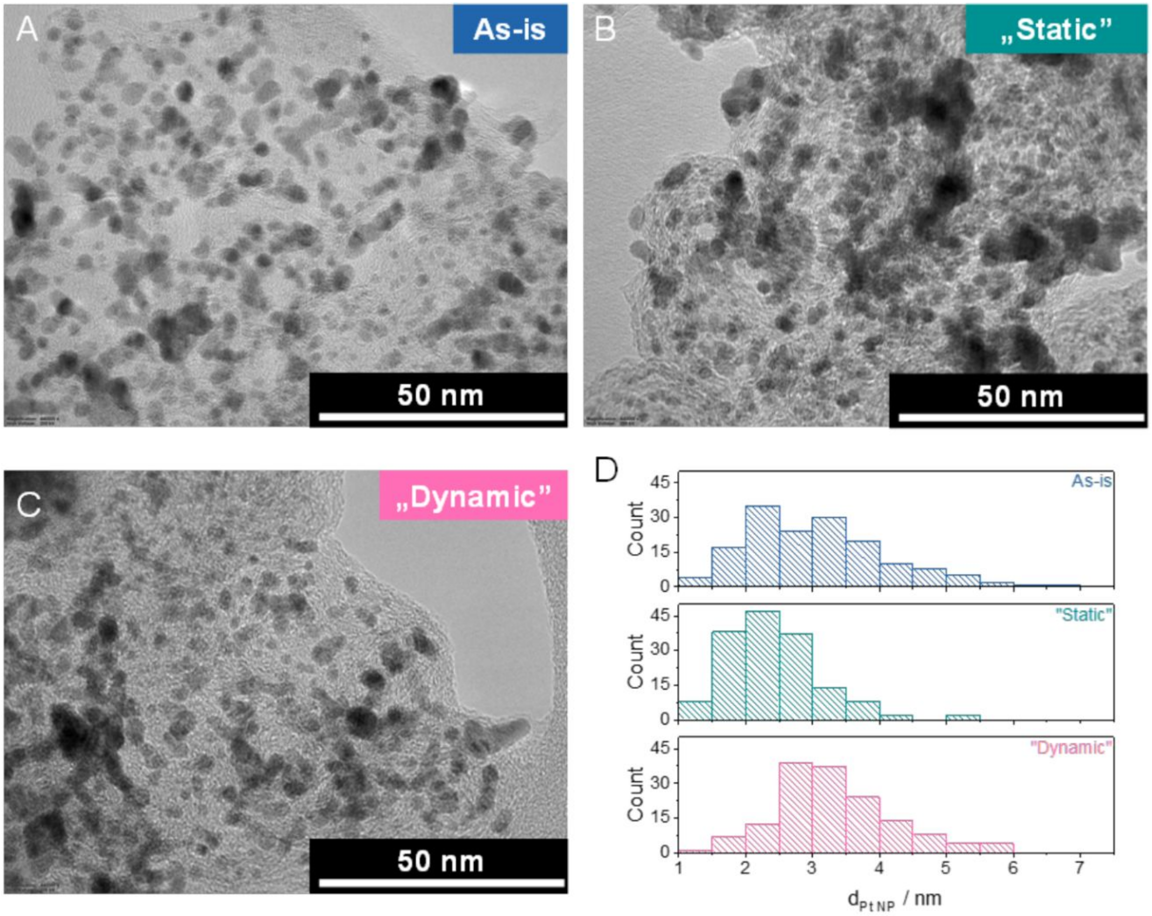

Figure S14. (A)-(C) TEM images captured for the pristine Pt/C+5 wt% CST catalyst layer and for the catalyst layers after performing either the "static" or "dynamic" long-term electrochemical protocols. (D) Pt nanoparticle size distribution calculated from the TEM data in A-C measuring the diameter of 150 nanoparticles.

References

(1) Fatemeh, K.; Mohammad Javad, M.; Samaneh, K. The Effect of Silver Nanoparticles on Composite Shear Bond Strength to Dentin with Different Adhesion Protocols. *J. Appl. Oral Sci.* **2017**, *25* (4), 367–373. https://doi.org/10.1590/1678-7757-2016-0391.

(2) Topalov, G.; Ganske, G.; Lefterova, E.; Schnakenberg, U.; Slavcheva, E. Preparation and Properties of Thin Pt–Ir Films Deposited by Dc Magnetron Co-Sputtering. *Int. J. Hydrogen Energy* **2011**, *36* (23), 15437–15445. https://doi.org/10.1016/j.ijhydene.2011.08.100.
